# Electrostatic potential and valence modulation in La_0.7_Sr_0.3_MnO_3_ thin films

**DOI:** 10.1038/s41598-018-32701-x

**Published:** 2018-09-25

**Authors:** Robbyn Trappen, A. C. Garcia-Castro, Vu Thanh Tra, Chih-Yeh Huang, Wilfredo Ibarra-Hernandez, James Fitch, Sobhit Singh, Jinling Zhou, Guerau Cabrera, Ying-Hao Chu, James M. LeBeau, Aldo H. Romero, Mikel B. Holcomb

**Affiliations:** 1 Department of Physics and Astronomy, West Virginia University, Morgantown, WV 26506 USA; 2 Physique Théorique des Matériaux, Université de Liège, B-4000 Sart-Tilman, Belgium; 3 Department of Physics, Universidad Industrial de Santander, Cra. 27 Cll. 9, Bucaramang, Colombia; 4 Institute of Physics, National Chiao Tung University, 30010 HsinChu, Taiwan; 5 Department of Mechanical & Aerospace Engineering, West Virginia University, Morgantown, WV 26506 USA; 6 Facultad de Ingeniería-BUAP, Apartado Postal J-39, Puebla, Pue. 72570 Mexico; 7 Department of Materials Science and Engineering, North Carolina State University, Raleigh, North Carolina 27695 USA

## Abstract

The Mn valence in thin film La_0.7_Sr_0.3_MnO_3_ was studied as a function of film thickness in the range of 1–16 unit cells with a combination of non-destructive bulk and surface sensitive X-ray absorption spectroscopy techniques. Using a layer-by-layer valence model, it was found that while the bulk averaged valence hovers around its expected value of 3.3, a significant deviation occurs within several unit cells of the surface and interface. These results were supported by first principles calculations. The surface valence increases to up to Mn^3.7+^, whereas the interface valence reduces down to Mn^2.5+^. The change in valence from the expected bulk value is consistent with charge redistribution due to the polar discontinuity at the film-substrate interface. The comparison with theory employed here illustrates how this layer-by-layer valence evolves with film thickness and allows for a deeper understanding of the microscopic mechanisms at play in this effect. These results offer insight on how the two-dimensional electron gas is created in thin film oxide alloys and how the magnetic ordering is reduced with dimensionality.

## Introduction

The family of materials known as manganites has received considerable attention in the last several decades as promising candidates for device applications like magnetic tunnel junctions^1^ and solid oxide fuel cells^2^. The widely-studied La_0.7_Sr_0.3_MnO_3_ (LSMO) is particularly appealing due to its properties such as large anisotropic magnetoresistance, high spin polarization, and above room temperature Curie temperature^1^. However, LSMO exhibits a problem that also exists in many other magnetic systems. When many magnetic materials are thin, their magnetic order is lost or reduced^3–5^. The reduction of magnetic order can also occur at the surfaces^6^ and interfaces^7^ of bulk materials which can be problematic for some applications. The layer of reduced or lost magnetism is called the magnetic dead layer.

Magnetism is not the only property that can change in thin films. The electrical conductivity of thin film LSMO is also reduced, exhibiting a temperature dependence consistent with insulators, typically below a thickness of 6 unit cells (u.c.)^8,9^. This thickness dependent metal-to-insulator transition has been shown to be a result of increased carrier scattering due to defects already present in the material exacerbated by the reduced dimensionality of the thin films^9^. While the loss of conductivity in thin films has been explained, the origin of the magnetic dead layer in complex oxides is still under debate. Density functional (DFT) calculations by Liao *et al*. show that LSMO should remain magnetic even at a thickness of 1 u.c.^9^ Studies have focused on factors such as strain^9,10^, oxygen defects^9,11^, and cation non-stoichiometry^11^. The influence of the polar LSMO/STO interface has also been studied^12–14^; however, the implications of this effect for the electronic structure of the material as well as its relation to other factors that influence the dead layer are not well-established. The dead layer problem restricts the development of devices that utilize effects such as magnetoresistance or interfacial magnetoelectricity, which require strong magnetism at the material boundary or interface; thereforethere is a strong need to systematically evaluate the parameter space of these materials in order to learn more about the origin of the MDL and how to work around it.

Material properties such as atomic valence are known to vary in thin film LSMO and may be related to the existence of the dead layer. While the end-parents of LSMO, La^3+^Mn^3+^O_3_^2−^ (with Mn^3+^) and Sr^2+^Mn^4+^O_3_^2−^ (with Mn^4+^) are both insulating antiferromagnetic materials, La_1−x_Sr_x_MnO_3_ is ferromagnetic for 0.1 ≤ *x* ≤ 0.5 due to the double exchange interaction^15^. Thus, the average valence, that is the Mn^3+^/Mn^4+^ ratio, plays a role in the magnetic and conductive properties of the material, which indicates that changes in valence values within the thin film may be related to the formation of the dead layer. The thickness dependence of the Mn valence has been previously studied by Shibata *et al*. who observed a sudden shift of the Mn *L*_3_ peak position for films with 6 u.c. thickness or less to lower energy, indicating a shift of the Mn valence toward 3+ ^8^. However, the study has pointed to the need for a more precise determination on how the valence and magnetism vary layer by layer within the material.

In this paper, we combine two X-ray absorption spectroscopy (XAS) techniques, one bulk and one surface sensitive, in order to experimentally determine how valence changes layer by layer within the material and compare with DFT calculations. This experimental approach has been previously used to study the layer-by-layer valence change in LSMO/PbZr_0.2_Ti_0.8_O_3_ (PZT) magnetoelectric heterostructures^16^. The surface charge of the ferroelectric PZT layer was found to be screened by the carriers in the LSMO layer, causing a change in the interfacial Mn valence depending on the ferroelectric polarization direction. Here we show how the Mn valence states, along the several LSMO layers, are modulated. We explain the source of such behavior where the electronic reconstruction induced by the polar layer leads to different orbital occupation per layer, which then results in a variation of the valence states per layer.

## Results and Discussion

La_0.7_Sr_0.3_MnO_3_ was deposited onto TiO_2_-terminated (001)-oriented SrTiO_3_ (STO) using pulsed laser deposition. Growth parameters are discussed in the Methods section^16^. Multiple thicknesses were measured within the range of 1–16 u.c.

**Figure 1:**
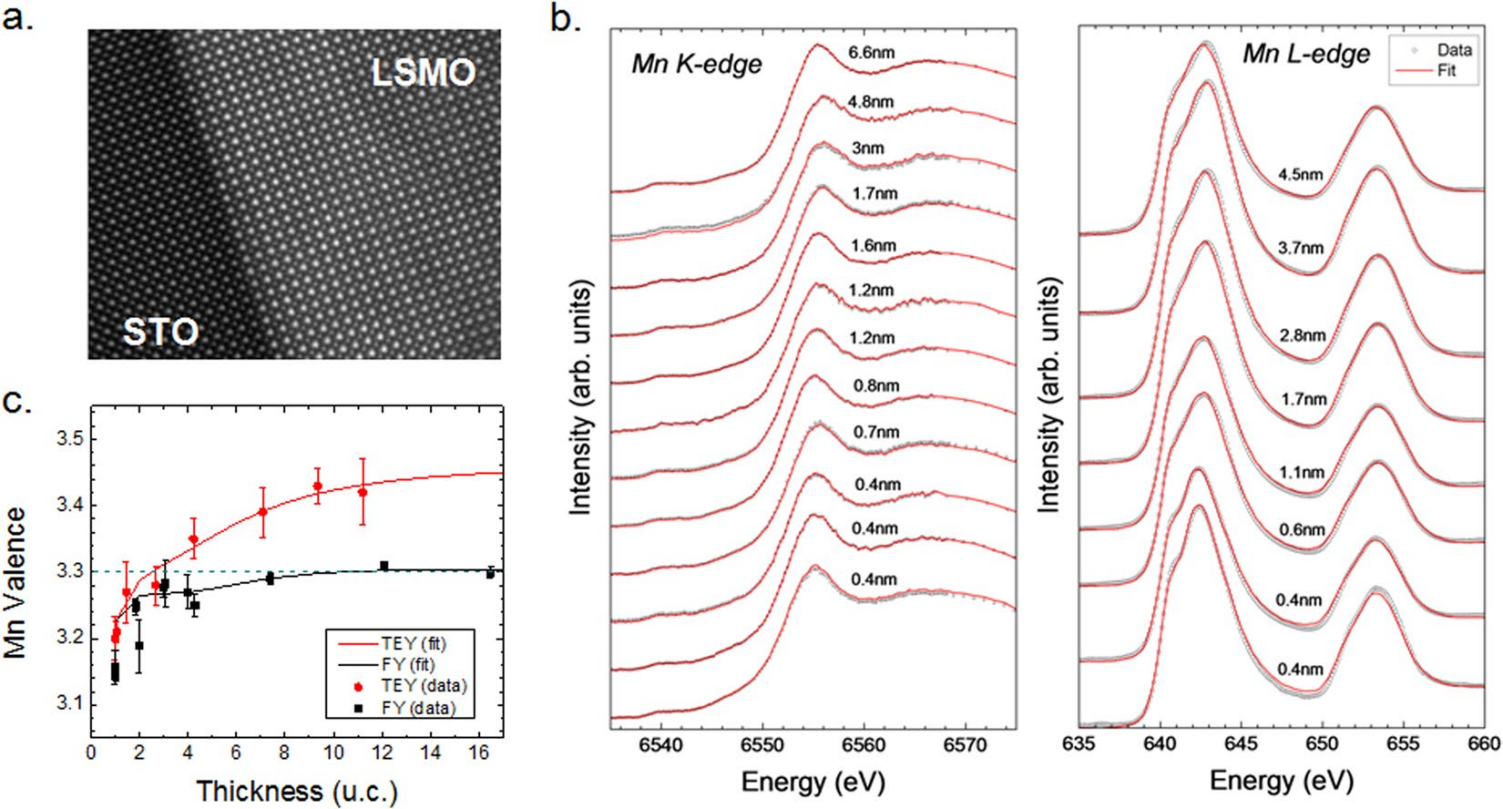
(**a**) TEM image of the film-substrate interface. (**b**) Results of the linear combination fits for the Mn K and L-edges. (**c**) Valence obtained from the linear combination fits (solid points) and experimental fit using the layer dependent valence model (solid lines). The dashed line indicates the expected bulk Mn valence of 3.3.

To determine sample and interface quality, transmission electron microscopy (TEM) measurements were performed. The details of sample preparation are discussed in the SI. Figure 1a shows the TEM characterization of the film-substrate interface, indicating an atomically sharp interface with no interdiffusion.

XAS measurements were performed to determine the thickness dependent change in the Mn valence via a combinatorial fitting approach. Measurement and fitting details are discussed in the methods section. The error bars were obtained by analyzing the variation between the valences obtained from fits using different combinations of references. Details are discussed in the supplemental information. In all cases, fits to the data, as shown in Fig. 1b, with near-noise residuals were obtained. Multiple fittings were performed, especially for some ultrathin samples (shown). Our results remained consistent for multiple measurements repeated over a period of one year. We note that the differences in the line shapes for the Mn K-edge spectra are not obvious from inspection of Fig. 1b, as differences between K-edge spectra can be subtle. This point is further discussed in the supplemental information.

Results of the linear combination fits to the XAS are presented in Fig. 1c. The Mn valence of bulk LSMO (dashed line), is 3.3 due to the sample composition of 70% LaMnO_3_ (Mn^3+^) and 30% SrMnO_3_ (Mn^4+^). As can be seen, the FY valence is close to the bulk value above a thickness of 4 u.c. Below this thickness, the measured valence begins to decrease. The TEY valence is above 3.3 for thicknesses greater than 4 u.c. indicating that the surface valence is larger than the bulk value. The smooth transition of valence at low thicknesses seem to somewhat contrast the results of Shibata *et al*. who observe an abrupt shift of the absorption edge from bulk-like behavior toward 3+ below a critical thickness of 6 u.c. The discrepancy might be due to a cap layer of La_0.6_Sr_0.4_TiO_3_ in their samples because capping layers have been reported to affect the Mn valence at the surface^17,18^. As FY provides a near average of the material properties due to the large probing depth, the decrease in the Mn valence below 4 u.c. suggests a change in the overall average valence of the material. One mechanism that can explain this change is the presence of oxygen vacancies, which are known to lower the Mn valence in LSMO by the formation of Mn^2+^ sites^19^. It is not surprising that films of a few unit cell thickness would be more sensitive to the formation of oxygen vacancies, due to the overall smaller number of atoms in the layer, i.e. removal of a given number of oxygen atoms in a 1 u.c. film results in a more noticeable effect on the Mn valence than in say an 8 u.c. film. Additionally, a greater percentage of the layer volume is in contact with vacuum/air in this case due to the reduced thickness, and oxygen vacancy formation may be more likely due to the chemical potential gradient between the film and air, as has been noted in other works^19–21^.

**Figure 2:**
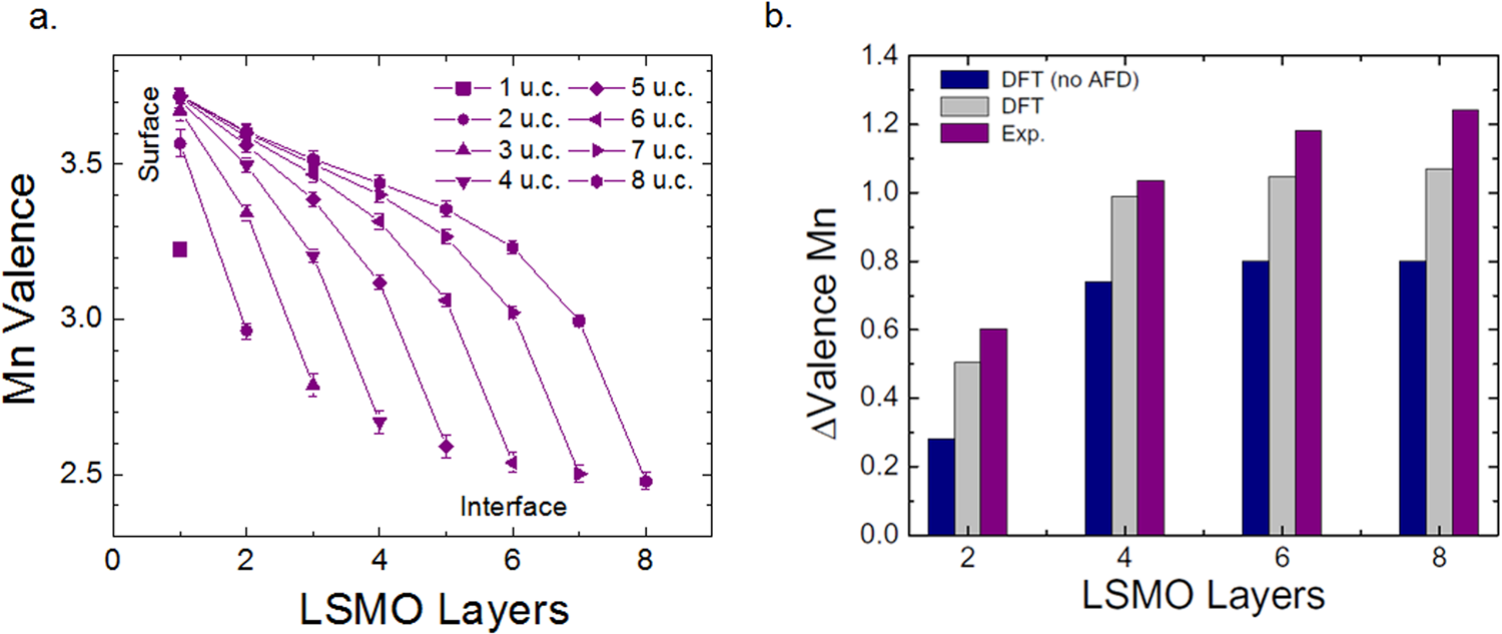
(**a**) Plane-resolved valence from the experimental fit. (**b**) Valence difference at the Mn-sites between the surface and the interface for the 2, 4, 6, and 8 u.c. of LSMO obtained from the DFT with and without antiferrodistortive (AFD) rotations and experiment.

To better understand the observed thickness-dependent change in valence, we fit both FY and TEY data using a layer-dependent valence model developed in a previous work^16^. The fit to the experimental data is shown in Fig. 1c (solid lines) and the layer-by-layer valence extracted from the fits is shown in Fig. 2a. Our experimental results indicate a raised surface valence that approaches 3.7 for the thicker films and a lowered interface valence of 2.5. Additionally, the net variation of the valence state is LSMO thickness dependent, thus, for thinner films, close to 2 u.c., the modulation from the surface to the interface is close to 0.6 in contrast to 1.2 for the 8 u.c. films. Our results are consistent with several transmission electron microscopy studies^13,22,23^ where a drop in the Mn valence near the interface was observed.

We note that the valence for ultrathin LSMO (1–2 u.c.) seems to deviate from the observed trend in Fig. 1c. As discussed earlier, thin LSMO is known to have different properties from thicker films, losing its magnetism and metallicity, so this result is not entirely surprising. Despite the deviation, we note that the difference between the surface and interface valence agrees well with the DFT results (see below). To determine the effect of these data points on the profile obtained from the experimental fit, we ran the fit both with and without the outliers in the data set. We observed that removing these data points from the data set and rerunning the fit only changed the fit values *V*_*thin*_ and *V*_*int*_ by approximately 0.04 (see S.I.). In addition, the layer-by-layer valence determined from the fit (Fig. S3) is almost identical to the trend obtained by including the observed valence in the 1–2-unit cell thick films. Therefore, the model used here is robust against variation in the thin data.

To gain further insight into the microscopic details of the observed change in valence, DFT calculations were performed. As discussed in more detail in the supplemental section, we have built a computational slab, where the STO is TiO_2_ terminated and therefore, the (La/Sr)O layer lies on top of this layer. After a full electronic and structural relaxation, we have determined the valence state for the Mn-sites at each layer for thin-films of different thicknesses. During our initial calculations, we observed while the Mn valence of our buried layers got smaller near the interface, the valence at the surface layer exhibits an abrupt drop in full disagreement with the experimental results. This drop is explained in terms of the uncompensated MnO_2_ at the surface. We found that an oxidation of the surface, which completes the surface octahedron, is necessary to compensate the potential and obtain consistent values of the valence state at the surface layer as compared with experiment. This result is reasonable since the presence of oxygen at the surface will result in more electrons from the surface Mn atoms being bonded with their neighbors – fewer electrons on the Mn sites will result in a larger overall Mn valence, and the absence of the surface oxygen atoms leads to fewer electrons participating in bonding and thus a lower valence. As our oxygen pressure is increased at the end of growth until the sample is cooled to discourage oxygen desorption, a completed oxygen octahedron is not surprising. From now on, all our results below will refer only to this situation of fully terminated oxygen octahedron at the surface.

Figure 2b summarizes the net valence difference between the Mn-sites at the surface and those at the interface, obtained from the DFT and the experimental measurements. It is shown, for example, that in the 4 u.c. films we obtain a decrease close to 1.0 e- of the Mn valence as we move from the surface’s MnO_2_ plane to the same plane at the interface. The trend followed by this charge difference is saturated to a value of 1.1 (see Fig. 2b) which is close to the expected valence value obtained experimentally. As can be concluded from this figure, the charge valence difference between surface and interface changes from 0.6 e- to 1.2 e- as the thickness increases from 2 u.c. to 4 u.c. The differences between experiment and theory may be explained by the presence of oxygen vacancies, which are not taken into account in our calculations. The presence of oxygen vacancies has been seen to lower the Mn valence at the film-substrate interface^23^, which may enhance the valence difference between the surface and the interface compared to theoretical predictions.

Regardless of whether oxygen vacancies are present or not, however, the overall change in valence across the film is still greater than the expected value of 1 e- predicted by the polar catastrophe model. In order to explain this trend, the mechanism of octahedral rotation must be considered. The effect of antiferrodistortive (AFD) rotations is illustrated in Fig. 2b. Here, the valence change across the film is compared for the case of a structure with and without AFD. As can be seen, the AFD greatly enhances the charge transfer from the surface to the interface. This is because the rotations increase the Mn-O orbital overlap, leading to increased charge transfer between sites, similar to earlier results reported for the LAO/STO system^24,25^. For the case of no AFD, the charge transfer remains below 1 e- as predicted by the simpler polar catastrophe model, but in disagreement with the experimental results.

**Figure 3:**
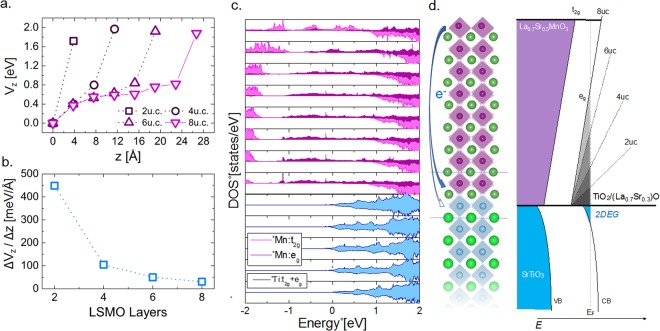
(**a**) The behavior of the electrostatic potential per layer as a function of the number of LSMO layers deposited. (**b**) Averaged changes in the electrostatic potential per thickness for samples with different number of LSMO layers deposited. (**c**) Orbital and layer resolved DOS for 8 layers of LSMO. (**d**) Schematic representation of the band alignment and charge transfer phenomena as a function of LSMO film thickness. Vertical segmented line represents the Fermi level, the blue filled line represents the first conduction band of STO while the gray filled lines represent states from LSMO layers. Different dashed lines represent the slope of the electrostatic potential for different LSMO-layers deposited. Finally, blue arrows depict the charge transfer from the surface to the interface.

In order to offer additional insight into the dependence of the Mn valence as function of the number of LSMO layers, we have computed the layer dependent electric potential and averaged changes in the electrostatic potential per thickness for different LSMO film thicknesses (Fig. 3a,b). The behavior of the potential illustrated in these figures show that the electrostatic potential is highly non-linear and the variation is larger close to the surface and interface as shown in Fig. 3a. The trend is in good agreement with the experimental data, where the valence changes are more pronounced at the interface and the surface, while there is a steady change in the middle of the LSMO; however, it is noted that the calculations indicate that the potential changes more rapidly at the surface than the interface, in contrast to the experimental results. The presence of oxygen vacancies at the interface likely provides an additional contribution to this trend as the positive charge of the vacancies will lead to more electrons accumulating at the interface in order to neutralize the charge and likely lead to a larger potential difference across the interfacial layers than would be expected otherwise.

Fig. 3b depicts the averaged changes in the electrostatic potential across the film thickness, extracted by fitting the layer dependent potential from Fig. 3a to a linear function and neglecting the surface contribution. The value of this change, close to 450 meV/Å at the 2 u.c. thin-film, is due to the presence of the charged-layers, [(La_0.7_Sr_0.3_)O]^0.7+^ and [MnO_2_]^0.7−^. As discussed below, the polar-catastrophe phenomenon is the source of the observed electrostatic potential. As this material is metallic in one of the spin channels (see band structure in the S.I. as well as the DOS in Fig. 3c), the charge is mostly accumulated from the gapped spin channel. This potential is then created by the system with the aim to compensate the disproportionate charge between surface and interface^26^. Consequently, as expected in a half-metallic system, a charge-transfer takes place in order to mitigate the large potential and with it, the transition metal cation valence charge will be modulated under the effect of this potential. Therefore, as the thickness increases, the electric field is smaller due to the transferred charge towards the interface. For thicknesses larger than 8 u.c. we still observe a half-metallic behavior of the LSMO with an insulating spin channel. Close to 8 u.c., the valence state comes together as a response of the electronic stabilization of the system and ΔV_z_/Δz converges to a single value independent of the number of layers close to 0 meV/Å. This process is schematically presented in Fig. 3d. Thus, the charge reconstruction has the effect of cancelling the diverging potential created by the polar catastrophe.

Additionally, the layer-by-layer DOS also confirms the formation of the electronic potential as shown by the DOS progressive displacement (from −2 to −1 eV) when moving from the interface to the surface. Analysis on the orbital contribution to the resolved layer-layer DOS shows a modulated occupation on the Mn:*e*_*g*_ levels as a result of the charge transfer, leading to an accumulation of carriers in the layers near the interface. The same analysis also shows that some charge is also transferred to the 3*d*:Ti states generating a 2DEG at the STO interface (also observed in the band structure in Fig. S5), though it shows a comparatively lower carrier density. We have computed the localized charge at the Ti atoms close to the interface and found carriers close to 6.0 × 10^12^ cm^−2^. This value is at least 2 orders of magnitude smaller as the one found at the LAO/STO and GTO/STO interfaces^25,27^.

Remarkably, the origin of this behavior is at least largely due to the charge reconstruction needed to prevent the polar catastrophe at the LSMO/STO interface^12,22^. Polar catastrophe has been seen in many other systems^27–29^ and one method to prevent the buildup of potential is to redistribute the electrons such that extra electrons are at the interface (as depicted in Fig. 3d). The extra electrons would lower the Mn valence at the interface^13,22^. Charge conservation would imply that because there is an accumulation of negative charge at the interface, there should be an overall positive charge left at the film surface. Moreover, the large electronic gap of the STO and the band alignment with LSMO causes the charge transfer to occur from the surface layer to the closest MnO_2_ layer to the in interface instead to the TiO_2_ plane as expected from the electronic reconstruction observed at the STO/LAO system.

## Conclusions

We have demonstrated consistent layer-by-layer calculation and measurement of the Mn valence across LSMO thin films. The experimental approach was to measure using both L-edge and K-edge absorption spectroscopy. By simultaneously globally fitting both sets of data, layer by layer valence was modeled and is in close agreement with theory. Theoretical efforts utilized first principles DFT calculations. Our results indicate that the Mn valence changes significantly near the LSMO/STO interface layer. This valence modulation is driven by the polar-catastrophe phenomenon which happens in a single spin channel and that is at the same time, modulated by the number of LSMO layers.

The approach of using two measurement techniques—here both synchrotron absorption techniques—with different probing depths (in combination with theory) is broadly applicable to studying changes in material properties with depth. As many applications call for the need for thinner materials, these proximity effects will become increasingly important. In the case of LSMO, this combined approach could be particularly powerful if combined with systematic changes of material properties (such as oxygen vacancies or strain) to probe the strongly correlated nature of these materials. Such efforts may help determine the origin of and how to possibly eliminate the dead layer behavior. In addition, the understanding of proximity effects may allow the enhancement and control of interfacial properties beyond those possible in bulk.

## Methods

The Mn K-edge and L-edge measurements were taken at ALS beamlines 10.3.2 and 6.3.1 respectively. K-edge spectra were taken in fluorescence yield (FY) mode and L-edge spectra were measured in total electron yield (TEY) mode. All spectra were normalized to the incident x-ray flux. Spectra were treated by subtracting the pre-edge background by fitting to a power law and then normalizing the post-edge to 1. The Mn valence for both the K and L-edge were determined by fitting each spectrum with a linear combination of reference spectra^30,31^. This combinatorial approach^30^ was developed for the Mn K-edge and has here been applied to the Mn L-edge as well, with a two-arctangent step function correction^31^. The K-edge reference dataset was taken from reference^30^ while the Mn L-edge references were digitalized from the spectra in references^32–37^.

Our films were fabricated by pulsed laser deposition with assisted high pressure reflection high-energy electron diffraction (RHEED). A KrF excimer laser manufactured by Lambda Physik was used with wavelength 248 nm at a laser energy of 300mJ/pulse and repetition rate of 10 Hz. LSMO samples were prepared on treated STO single crystals (The TiO_2_ terminated STO (100) surfaces were obtained by chemical treatment with an HF-NH_4_F buffer solution). We used *in-situ* RHEED to monitor layer growth of LSMO. Films were deposited at growth temperatures of 700 °C and oxygen pressures of 80 mTorr. Following layer deposition, full oxygenation was achieved by annealing the film at 550 °C in an oxygen atmosphere of 700 Torr for an 20 min followed by slow cooling to room temperature.

## Electronic supplementary material

Supporting information

## Data Availability

The datasets generated during and/or analyzed during the current study are available from the corresponding author on reasonable request.
