# Supplementary material for: Electrostatic potential and valence modulation in La0.7Sr0.3MnO3 thin films
Source: Sci Rep. 2018 Sep 25;8:14313. doi: 10.1038/s41598-018-32701-x (PMC6156561; doi:10.1038/s41598-018-32701-x)
Supplement: Supplementary file 1 — Supporting information [file 41598_2018_32701_MOESM1_ESM.doc]

**Supporting information for “Electrostatic potential and valence modulation in La_0.7_Sr_0.3_MnO_3_ thin films”**

Robbyn Trappen*^,1^, A. C. Garcia-Castro^1,2,3^, Vu Thanh Tra^4^, Chih-Yeh Huang^5^, Wilfredo Ibarra-Hernandez^1,6^, James Fitch^7^, Sobhit Singh^1^, Jinling Zhou^1^, Guerau Cabrera^1^, Ying-Hao Chu^4^, James M. LeBeau^7^, Aldo H. Romero^1,6^ and Mikel B. Holcomb*^,1^

*^1^Department of Physics and Astronomy, West Virginia University, Morgantown, WV 26506, USA*

*^2^Physique Théorique des Matériaux, Université de Liège, B-4000 Sart-Tilman, Belgium*

*^3^Department of Physics, Universidad Industrial de Santander, Cra. 27 Cll. 9, Bucaramang, Colombia.*

*^4^Institute of Physics, National Chiao Tung University, 30010 HsinChu, Taiwan*

*^5^ Department of Mechanical & Aerospace Engineering, West Virginia University, Morgantown, WV 26506, USA.*

*^6^Facultad de Ingeniería-BUAP, Apartado Postal J-39, Puebla, Pue. 72570, México*

*^7^Department of Materials Science and Engineering, North Carolina State University, Raleigh,*

*North Carolina 27695, USA*

***I. Discussion of the lineshapes of the Mn K-edge spectra***

Differences between Mn K-edge spectra are in general more subtle than those in the L-edge. This is largely due to the presence of multiplet features in the L-edge which make one spectrum more readily distinguishable from one another. Nevertheless, close inspection of the spectra reveals some distinct differences between the various cases.

Figure S1 shows two spectra from thin and thick LSMO (1 and 16.5u.c. respectively) in the top panel and their derivatives as a function of energy in the bottom panel. A reference from bulk LSMO is plotted for comparison. Several differences are apparent here. First is that the position of the most prominent peak in the spectra differ by about 0.4 eV (approx. 6555.0 eV and 6555.4 eV for thick LSMO). This can also be seen in a shift of the maxima of the first derivate in the bottom panel.


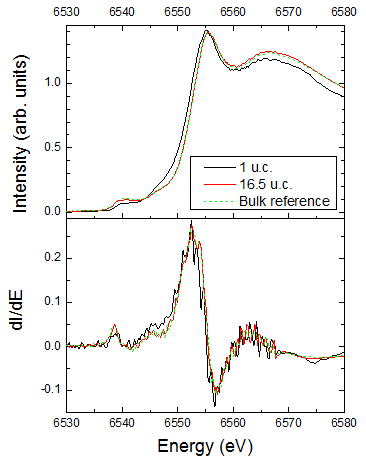


*Figure S1. Comparison of the Mn K-edge XAS (top panel) and the derivative of the spectra (bottom panel) for 1 u.c. (black), 16.5u.c. (red), and bulk LSMO (green, dashed).*

The 16.5u.c. spectrum, for which the Mn valence was determined to be 3.3 by fitting, almost exactly matches the spectrum for bulk LSMO, in which the valence is also 3.3. Additionally, some other features are evident on the lower energy side of the 1 u.c. data which lead to a somewhat broader peak structure as compared to the thick LSMO. Additionally, the quadrupole Mn 1s → 3d transition at 6540 eV is enhanced in the thicker spectra compared to the thin spectrum. The combinatorial fit is able to distinguish between these features *via* an appropriate combination of references, and as shown in the main text, the error from these fits was found to be very small.

***II. Valence uncertainty estimation***

One possible way to derive the error bars for the experimentally obtained valence is from the uncertainty in the fit parameters which would subsequently be fed into the propagation of error formula for the weighted average valence. Error bars obtained in this manner results in error bars of less than 1%. However, error bars derived in this way are only reflective of the uncertainty in the fit, that is, they give no indication on the *accuracy* of the valence estimate.

How do we know the true accuracy of the valence? Of course, if the combination of references completely reproduces the spectrum, we would expect the valence estimate to be very accurate. Likewise, if the references do not reproduce the spectral features, the estimate should be very poor. As a possible method of evaluating this accuracy, the mean squared error (MSE) was collected for the fit to each possible combination of references, as well as the valence for each fit. In principle, the most accurate fits should have the lowest MSE value and be clustered around some value of the valence which could be thought of as the true value and spread out away from this as the fit quality worsens.

A scatter plot of the MSE and valence from the fit for the 1 u.c. thickness (taken in TEY mode) is shown in Figure S2. The dashed line indicates the position of the valence obtained from the best fit. As can be seen, there is a cluster of points around the best fit value of 3.22 that spreads out as the MSE increases. Note that the MSE is plotted on a logarithmic scale, indicating that the points with higher MSE results represent much poorer fits. If there were more than one likely solution to the spectra, one would expect a very spread out set of solutions or multiple clusters with low error.


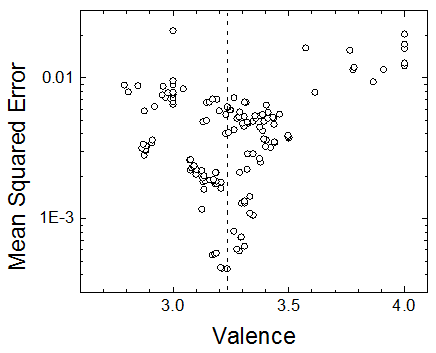


*Figure S2. Scatter plot showing the mean squared error vs. the valence obtained for the fit to the 1 u.c. TEY data.*

To evaluate the error bars, we wish to determine the spread of the good quality fits around the best value obtained with the references. The valence of the best fits (typically the lowest 5-10 which cluster around the lowest MSE) were averaged, weighted by the inverse of the MSE (i.e. to ensure that better fits contribute more strongly to the average) to determine the thickness dependent Mn valence reported in Figure 1b in the main text. The standard deviation of these best fits was obtained to give the error.

Additionally, the error from the fit itself (described above) should be taken into account as this too will affect the accuracy of the valence estimate. The valence from each linear combination fit is calculated as a weighted average by


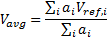


where *i* runs over all of the references used in the fit (a maximum of 4 were used here) and the
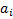
 are the coefficients for each of the references. These results were then input into the standard propagation of error formula along with the error from the fit parameters
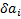
 as


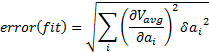


The error bars reported on the plots in the main text are then calculated as

*error(valence) = error(scatter plot) + error(fit)*

***III. Effect of outliers***

**
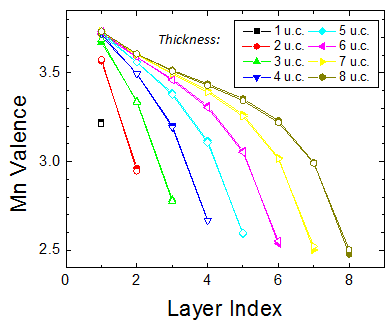
**

*Fig. S3. Comparison between the layer by layer valence obtained from the fit by including (solid points) and removing (open points) the 1-2 u.c. data that lie below average trend indicated by the fit in Figure 1c in the main text. Excluding these points results in very little difference to our resulting fit.*

***IV. Transmission electron microscopy characterization***

Samples for electron microscopy were prepared by conventional wedge polishing with an Allied High Tech Muli-prep polishing system. To achieve electron transparency, these samples were then argon ion milled (Fischione 1050) while maintaining the sample at liquid nitrogen temperature. A probe corrected FEI Titan G2 60-300 kV S/TEM equipped with an X-FEG source was operated at 200kV for STEM imaging and spectroscopy. The convergence semi-angle was 19.6 mrad. The HAADF images were corrected for drift using the RevSTEM method applied to a 40 frame 1024x1024 image series acquired using a dwell time of 1s.

***V. Computational details.***

As described in the text, we have performed ab-initio calculations in the framework of Density Functional Theory (DFT) as implemented in the Vienna Ab-Initio Simulation Package (VASP) [[[1]](#endnote-2),[[2]](#endnote-3),[[3]](#endnote-4),[[4]](#endnote-5)], where the pseudo-electron wave functions are described through the Projector-Augmented Wave (PAW) method [[[5]](#endnote-6),[[6]](#endnote-7)]. We have used a plane-wave energy cutoff of 550 eV to ensure good convergence of values for total energies and ionic forces. The exchange-correlation energy was described using the Generalized Gradient Approximation (GGA) with the Perdew-Burke-Ernzerhof exchange correlation functional revised for solids (PBESol) [[[7]](#endnote-8)]. The electronic configuration of elements pseudopotentials are: 10 electrons for Sr (4*s*^2^4*p*^6^5*s*^2^), 11 for La (5*s*^2^5*p*^6^5*d*^1^6*s*^2^), 12 for Ti (3*s*^2^3*p*^6^3*d*^2^4*s*^2^), 13 for Mn (3*p*^6^4*s*^2^3*d*^5^) and 6 for O (2*s*^2^2*p*^4^). We have used the LDA + U approximation within the Liechtenstein formalism to increase the localization of d-electrons in Mn atoms. The values of U and J that we have imposed are 2.7 and 1.0 eV, respectively. We have discretized the reciprocal space with a Monkhorst-Pack k-point grid of 8×8×1 for the thin-films and 6×6×4 for the bulk structures with 20 atoms per unit cell. The thin-film structure consists of 5 cubic layers of SrTiO_3_ (STO), where we have fixed the first layer to simulate the bulk while the remaining 4 layers and those of La_0.7_Sr_0.3_MnO_3_ (LSMO) were set free to relax. On top of the 5 layers of STO, we have put 2, 4, 6 and 8 layers of LSMO and we have included extra oxygen atoms at the surface to complete the Mn octahedron. To avoid artificial effects caused by the interaction with periodic images, we have imposed a 16 Å vacuum perpendicular to the LSMO surface.We have performed atomic relaxation until the forces were no larger than 1meV/Å. We have used the virtual crystal approximation (VCA) to model Sr doping [[[8]](#endnote-9)].

***VCA reliability in VASP.***

Even though previous theoretical calculations have shown that the use of VCA in VASP correctly reproduce lattice parameters and experimental magnetic moments in LSMO [[[9]](#endnote-10)], we have performed our own calculations to test the VCA model implemented in the VASP code. We compute the structural and electronic properties of La_0.5_Sr_0.5_MnO_3_ and compared the results obtained with VCA. Different distributions of Sr and La over all possible A-sites in a
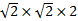
(with respect to the pseudocubic) Pnma structure with 20 atoms were considered. We looked at the lattice parameter of the pseudo-cubic system as well as the electronic structure and calculated band-gap. With respect to the lattice parameters, we have found that VCA method gives smaller values than the structure with alternate Sr and La atoms occupying the A-sites. The layer difference that we have obtained is just 0.47%. With respect to the electronic structure, our calculations show that the structure with alternate atoms in the A-site has a band-gap for spin-down of 2.30 eV while the gap found with VCA method the gap is 2.45 eV. The calculations of Moreau *et al*. show the same increase in the band-gap of VCA with respect to the built-instructures. Ultimately, the Mn magnetic moment shows a difference of only 0.01μ_B_ between the two cases. With all these and the mentioned reference, we conclude that the results obtained with VCA in the VASP code are adequate in regards to structural, electronic and magnetic properties for the LSMO alloy.

|  |
| --- |
| *Figure S4. Electronic density of states (DOS) for the structure with alternate Sr and La atoms in the A-sites (top panel) and for the one obtained with VCA method (bottom panel). We also show the partial DOS for d-orbitals of Sr, La and Mn as well as for p-orbitals of oxygen.* |

***VI.*** Electronic band structure of 8 layers of LSMO.

| 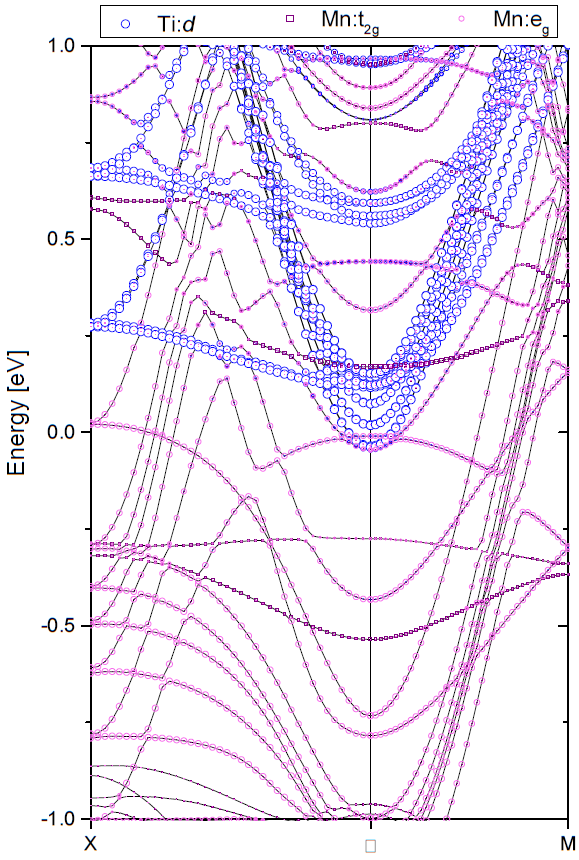  Γ |
| --- |
| *Figure S5. Electronic band structure of obtained from the thin-film with 8 layers of LSMO on top of 5 layers of STO. The bands show the parabolic band coming from Ti d-orbitals.* |

***VII. Calculation of the electrostatic potential***

In order to obtain the electrostatic potential reported in Figure 3a in the main text, the 3D local electronic potential is computed including the exchange correlation part. Later, an integration along the *z*-axis is performed and a moving average, of one unit-cell window, is applied.

1. . Kresse, G. & Hafner, J. Ab initio molecular dynamics for liquid metals. *Phys. Rev. B* **47**, 558 (1993). [↑](#endnote-ref-2)
2. . Kresse, G. & Hafner, J. Ab initio molecular-dynamics simulation of the liquid-metal–amorphous-semiconductor transition in germanium. *Phys. Rev. B* **49**, 14251 (1994). [↑](#endnote-ref-3)
3. . Kresse, G. & Furthmüller, J. Efficiency of ab-initio total energy calculations for metals and semiconductors using a plane-wave basis set. *Comp. Mater. Sci.* **6**, 15 (1996). [↑](#endnote-ref-4)
4. . Kresse, G. & Furthmüller, J. Efficient iterative schemes for ab initio total-energy calculations using a plane-wave basis set. *Phys. Rev. B* **54**, 11169 (1996). [↑](#endnote-ref-5)
5. . Blöchl, P. E. Projector augmented-wave method. *Phys. Rev. B* **50**, 17953 (1994). [↑](#endnote-ref-6)
6. . Kresse, G. & Joubert, D. From ultrasoft pseudopotentials to the projector augmented-wave method. *Phys. Rev. B* **59**, 1758 (1999). [↑](#endnote-ref-7)
7. . Perdew, J. P. *et al.* Restoring the Density-Gradient Expansion for Exchange in Solids and Surfaces. *Phys. Rev. Lett.* *100*, 136406 (2008). [↑](#endnote-ref-8)
8. . Bellaiche, L. & Vanderbilt, D. Virtual crystal approximation revisited: Application to dielectric and piezoelectric properties of perovskites. *Phys. Rev. B* **61**, 7877 (2000). [↑](#endnote-ref-9)
9. . Moreau, M. Selbach, S. M. & Tybell, T. Octahedral coupling in (111)- and (001)-oriented La_2/3_Sr_1/3_MnO_3_/SrTiO_3_ heterostructures. arXiv:1710.10996 [cond-mat.mtrl-sci] [↑](#endnote-ref-10)
